# Description of 3 failed attempts to estimate the calcium equivalency of phytase for growth performance and tibia ash of broiler chickens when using graded dietary levels of limestone

**DOI:** 10.1016/j.psj.2023.103330

**Published:** 2023-11-28

**Authors:** Carrie L. Walk, Opeadura T. Osunbami, Olayiwola Adeola

**Affiliations:** ⁎ DSM Nutritional Products, Kaiseraugst, Switzerland; † Purdue University, West Lafayette, IN, USA

**Keywords:** calcium, direct method, equivalence, indirect method, phytase

## Abstract

Three broiler experiments were conducted to estimate the Ca equivalency of a novel phytase using direct and indirect methods. All 3 experiments employed 4 concentrations of limestone to create 4 reference diets, deficient in nonphytate P, with increasing dietary Ca. Phytase was supplemented to the lowest Ca reference diet at 350, 700, 1,400, or 2,800 FYT/kg in experiment (**Exp.**) 1 and Exp. 2 and at 500, 1,000, 2,000, or 4,000 FYT/kg in Exp. 3. Broilers were fed from d 8 to 10 and 20 to 24, 19 to 21, or 7 to 10 and 7 to 21 posthatching in Exp. 1, 2, or 3, respectively. Diet did not affect growth performance or tibia ash in Exp. 1. Reducing the dietary Ca linearly (*P* < 0.05) increased body weight gain (**BWG**) and feed intake (**FI**) in Exp. 2 or Exp. 3. Feed conversion ratio (**FCR**) was decreased (linear or quadratic, *P* < 0.05) as dietary Ca was reduced in Exp. 2 or Exp. 3 (d 7–21). Tibia ash percent linearly (*P* < 0.05) decreased as dietary Ca decreased in Exp. 3 but only from d 7 to 21 and phytase increased (linear or quadratic, *P* < 0.05) FI and BWG, and decreased FCR. In Exp. 1 (d 8–10) and Exp. 2, apparent ileal digestibility (**AID**), total tract retention, and apparent digested and retained Ca or *P* increased (linear or quadratic, *P* < 0.05) as dietary Ca decreased. Phytase increased (linear or quadratic, *P* < 0.05) AID and apparent digested and retained Ca or *P* in Exp. 1 or Exp. 2. Due to the nature of the effect of dietary Ca on performance or tibia ash, it was not possible to use the indirect method to estimate the Ca equivalence of phytase in the current experiments. The total and digestible Ca equivalence of phytase could be estimated using the direct method. These experiments highlight challenges to consider when designing experiments to estimate the Ca equivalency for phytase in the future.

## INTRODUCTION

Plant-based cereals and protein meals commonly used in poultry diets contain from 0.21 to 1.65% total P (Aureli et al., 2017), with approximately 50 to 60% stored in the form of phytate (Tahir et al., 2012). Phytate is digested by poultry, but at variable concentrations due to animal age, species, and dietary Ca (Sommerfeld et al., 2018; Novotny et al., 2023). Once ingested, phytate may chelate with minerals such as Ca, Zn, Fe, or Mg, amino acids, and protein (Ravindran et al., 2006) and can interfere with endogenous enzyme efficacy (Liu et al., 2008; Yu et al., 2012). In this case, phytate is considered a significant antinutrient in poultry diets. Exogenous phytase enzymes are supplemented into poultry diets to degrade phytate, improve nutrient utilization (Ravindran et al., 2006), and in some cases growth performance through the removal of the antinutritional properties of phytate (Walk et al., 2013).

Studies to estimate nutrient release values of phytase, also known as nutrient equivalence studies, employ 2 common experimental methods: the direct and the indirect method (Dersjant-Li et al., 2019). The direct method provides digestible or retained nutrient improvement values. It relies on in vivo studies, practical diets, an inert marker or total feces collection, and graded doses of phytase added to a nutrient-deficient control diet. The response criteria include apparent ileal digestibility (**AID**) or apparent total tract retention (**ATTR**). The nutrient equivalence of phytase is estimated from the phytase-related improvement in the AID or ATTR of a nutrient above that of the nutrient-deficient control diet. In contrast, the indirect method is used to estimate the nonphytate P (**nPP**), available P (**avP**) or the digestible P (**dgP**) equivalence of phytase compared with an inorganic source of P, such as monocalcium or dicalcium phosphate. The indirect method relies on regression analyses using growth performance and tibia ash, incremental increases in the inorganic phosphate source to create deficient to adequate P-levels, and graded doses of phytase added to the diet with the lowest dietary P concentration (Adedokun et al., 2004; Adeola, 2010).

In general, the direct method can be used to estimate the dgP, Ca, mineral, and amino acid release values for phytase. However, due to the adaptation of broilers to mineral-deficient diets, an increase in nutrient digestibility may be noted in the nutrient-deficient control diets, which will result in an underestimation of the phytase equivalence. When using the direct method, it is recommended to feed the test diets for less than 48 h (Li et al., 2018; Walk et al., 2023). The estimated nutrient equivalence of phytase can be influenced greatly by the digestibility of the control diet. Factors that may influence the control diet digestibility include, dietary Ca, phytate, P and broiler age and intake, and length of time on the study diets (Li et al., 2018; Babatunde et al., 2019a, 2019b; Walk et al., 2023). Finally, due to the short-term nature of the direct studies, growth performance and tibia ash are rarely evaluated as response criteria. In contrast, the indirect method is useful because it considers both growth performance and skeletal integrity (bone ash) as response criteria, is usually conducted for a minimum of 7 d, and can provide an estimate of nPP, avP, or dgP release values. However, to date, the indirect method is only applicable to P equivalence values for phytase (Adedokun et al., 2004; Adeola, 2010) and is rarely used to estimate other nutrient release values for phytase, such as minerals and amino acids.

Due to a high propensity for Ca and phytate to interact within the gastrointestinal tract of poultry, the release of Ca from phytate by phytase is presumed to be greater than that of P. In 1968, Nelson et al. estimated that the total Ca requirement of broilers could be reduced in the absence of dietary phytate. More recently, Selle et al. (2009) described the phytate molecule as carrying a maximum of 12 negative charges and could potentially chelate 6 Ca atoms, particularly because Ca^2+^ is the dominant cation in poultry diets. However, the influence of phytase on Ca digestibility and subsequent direct estimations of Ca release values for phytase are inconsistent. For example, Ravindran et al. (2006) or Li et al., (2021) reported significant improvements in the AID of Ca from phytase. In contrast, Davin et al. (2020) and Zhang et al. (2022) found the AID of Ca decreased with increasing phytase doses when using the direct method and long-term feeding studies. Therefore, the objective of this work was to compare the indirect and direct methods to estimate the Ca equivalence of a novel microbial phytase, including graded concentrations of Ca from limestone, different experimental methods, and graded doses of phytase in broilers at different posthatch ages.

## MATERIALS AND METHODS

Experiments (**Exp**.) 1 and 2 were conducted at DSM Nutritional Products research facilities in Village Neuf, France. The animal protocol for the studies was approved by the Ethical Committee of DSM Animal Nutrition Research Center and complied with the guidelines in the European Union Council Directive 2010/63/EU for animals used for experiments. Exp. 3, conducted at the Purdue University Animal Sciences Research and Education Center, West Lafayette, IN was reviewed and approved by the Institutional Animal Care and Use Committee under protocol number 1311000983.

### Experiment 1

#### Animals and Experimental Diets

Male Ross 308 chicks were obtained on d 0 posthatching and randomly allocated to battery cages in an environmentally controlled room with a lighting program of 12L:12D for the duration of the trial. Feed and water were offered for ad libitum consumption. A nutrient adequate common starter diet was fed from d 0 to 7 and again from d 11 to 20 posthatching. On d 8, birds (704) were assigned to 8 experimental diets using a randomized complete block design replicated 8 times with 11 birds per replicate. Four diets were formulated to contain graded concentrations of standardized ileal digestible (**SID**) Ca at 5.2, 4.2, 3.2, or 2.2 g/kg from d 8 to 10 or 4.6, 3.6, 2.6, or 1.6 g/kg from d 20 to 24 (Table 1). Nonphytate P in all diets was formulated at 2.0 g/kg from d 8 to 10 or 1.4 g/kg from d 20 to 24. Phytase was added to the diets with the lowest SID Ca at 350, 700, 1,400, or 2,800 FYT/kg. The phytase was a novel phytase, encoded by a 6-phytase gene from *Citrobacter braakii* with improvement in intrinsic temperature and pH stability (HiPhorius, DSM Nutritional Products, Kaiseraugst, Switzerland). All 8 diets were formulated to be isoenergetic and isonitrogenous at metabolizable energy and crude protein of 3,000 kcal/kg and 230 g/kg from d 8 to 10 and 3,100 kcal/kg and 215 g/kg from d 20 to 24. All diets contained 1.0 g/kg titanium dioxide as an inert marker for the determination of AID and ATTR of Ca and P.

**Table 1:** Ingredient and calculated nutrient content of the diets fed in experiment 1 and 2 on an as fed basis.

	Experiment 1 (d 0 to 10)			Experiment 1 (d 11 to 24)			Experiment 2 (d 0 to 21)		
		Experimental diets (d 8 to 10)		Common grower diet (d 11 to 19)	Experimental diets (d 20 to 24)		Common starter diet (d 0 to 18)	Experimental diets (d 19 to 21)	
Ingredient, g/kg	Common starter diet (d 0 to 7)	5.2 g/kg SID Ca	2.2 g/kg SID Ca		4.6 g/kg SID Ca	1.6 g/kg SID Ca		5.2 g/kg SID Ca	2.2 g/kg SID Ca
Wheat	596.5	612.9	638.3	622.2	638.9	664.2	–	–	–
Corn	–	–	–	–	–	–	557.3	569.5	596.1
Soybean meal	303.7	299.6	293.3	260	255.8	249.4	366.9	364.6	359.6
Rapeseed meal, sol. extracted	25	25	25	35	35	35	–	–	–
Soy oil	29.7	25.4	18.7	42.7	38.3	31.6	31.3	27.6	19.6
Salt	2.8	2.9	2.9	2.9	2.9	2.9	2.8	2.8	2.8
Limestone^1^	8.1	17.1	4.7	7.1	16.3	3.9	8.6	17.4	3.8
Dicalcium phosphate	21.2	3.2	3	18.5	0.1	–	22.5	6.4	6.4
Sodium bicarbonate	2	2	2	2	2	2	2	2	2
Lysine HCl	2.4	2.5	2.6	2	2.1	2.3	1	1.1	1.2
DL-methionine	2.9	2.9	2.9	2.5	2.5	2.5	2.9	2.9	2.8
Threonine	1.4	1.4	1.4	1	1.1	1.1	0.6	0.6	0.6
Premix^2^	4	4	4	4	4	4	4	4	4
Titanium dioxide	–	1	1	–	1	1	–	1	1
Protease^3^	0.05	0.05	0.05	0.05	0.05	0.05	0.05	0.05	0.05
Carbohydrase^4^	0.075	0.075	0.075	0.075	0.075	0.075	0.05	0.05	0.05
Formulated nutrient content, g/kg									
Crude protein	230.0	230.0	230.0	215.0	215.0	215.0	230.0	230.0	230.0
ME, kcal/kg	3,000.0	3,000.0	3,000.0	3,100.0	3,100.0	3,100.0	3,000.0	3,000.0	3,000.0
Dry matter	885.3	883.4	881.1	886.2	884.3	882.0	891.5	889.9	887.7
Total calcium	10.4	8.6	3.9	9.2	7.4	2.7	10.9	9.7	4.4
SID calcium	5.2	5.2	2.2	4.6	4.6	1.6	5.2	5.2	2.2
Available phosphorus	4.8	2.0	2.0	4.4	1.5	1.5	4.5	2.0	2.0
Digestible phosphorus	–	–	–	–	–	–	4.6	2.3	2.3
Total phosphorus	7.8	4.5	4.5	7.2	3.8	3.8	7.7	4.8	4.8
Nonphytate phosphorus	5.3	2.0	2.0	4.7	1.4	1.4	5.2	2.2	2.2
Phytate phosphorus	2.5	2.5	2.5	2.4	2.4	2.5	2.5	2.5	2.5
Digestible methionine + cysteine	9.5	9.5	9.5	8.7	8.7	8.7	9.5	9.5	9.5
Digestible lysine	12.8	12.8	12.8	11.5	11.5	11.5	12.8	12.8	12.8
Sodium	1.8	1.8	1.8	1.8	1.8	1.8	1.8	1.8	1.8
Chloride	2.9	3.0	3.0	2.9	3.0	3.0	2.9	3.0	3.0

1 The limestone used in experiment 1 had a GMD of 331 um, a total Ca of 388.8 g/kg, and an in vitro solubility (Kim et al., 2019) of 95.84 and 97.68% at 15 and 30 min, respectively. The estimated digestibility coefficient using the glycine buffer equation from Kim et al. (2019) was 0.648. The limestone used in experiment 2 had a GMD of 128 um, a total Ca of 387.0 g/kg, and an in vitro solubility (Kim et al., 2019) of 96.43 and 98.74% at 15 and 30 min, respectively. The estimated digestibility coefficient using the glycine buffer equation without phytase from Kim et al. (2019) was 0.567.

2 Vitamin and mineral premix provided (per kilogram of diet): vitamin A 10,000 IU; vitamin D_3_ 3,000 IU; vitamin E 40 mg; vitamin K_3_ 3 mg; vitamin B_1_ 2.5 mg; vitamin B_2_ 8 mg; vitamin B_6_ 5 mg; vitamin B_12_ 0.025 mg; biotin 0.15 mg; folic acid 1.5 mg; niacinamide 50 mg; D-pantothenic acid 12 mg; Fe (as FeSO_4_) 60 mg; Cu (as CuSO_4_) 15 mg; Mn (as MnO) 80 mg; Zn (as ZnO) 54 mg; I (as Ca(IO_3_)_2_) 1.2 mg; Se (as Na_2_SeO_3_) 0.297 mg; calcium as calcium carbonate (carrier) 26.4 mg.

3 The protease used in experiment 1 and 2 was ProAct 360 (DSM Nutritional Products, Kaiseraugst, Switzerland) with respective analyzed activity of 785,470 and 729,000 NFP/g, contributed 6.7 g/kg crude protein, 0.4 g/kg methionine + cysteine, 0.5 g/kg lysine, and 0.5 g/kg threonine.

4 The carbohydrase used in experiment 1 was Ronozyme WX 2000 CT (DSM Nutritional Products, Kaiseraugst, Switzerland) with an analyzed activity of 2,590 U/g and contributed 120 kcal/kg metabolizable energy. The carbohydrase used in experiment 2 was Multigrain (DSM Nutritional Products, Kaiseraugst, Switzerland) with an analyzed activity of 3,490 FXU/g and was added over the top of the basal diets.

#### Data Collection and Analysis

On d 8 and 10 posthatching, all birds were weighed and feed intake (**FI**) from d 8 to 10 was recorded based on the ad libitum feed consumption of 11 birds per cage. On d 10, 6 of the 11 birds per cage were randomly selected, stunned, and then euthanized by cervical dislocation. Digesta was collected on ice, by squeezing, and pooled per pen from the distal two-thirds of the ileum, defined as the Meckel's diverticulum to 40 mm proximal to the ileocecal junction. Excreta was collected daily from d 9 to 10 inclusive, pooled and homogenized within a cage, and frozen until further analysis. The remaining 5 birds per pen were fed a common, nutrient-adequate diet until experimental diets were fed again from d 20 to 24 of age. On d 20 and 24, all remaining birds were weighed and FI from d 20 to 24 was recorded. Excreta was collected from d 21 to 24. All birds were euthanized on d 24 and ileal digesta was collected as previously described. The right tibias were obtained from 2 birds per pen, pooled, and stored frozen. For the determination of tibia ash, tibias were stripped of adhering tissues, dried at 105°C for 24 h, and then ashed at 550°C for 48 h in a muffle furnace.

Digesta and excreta were freeze-dried to a constant weight. Diets, digesta, and excreta were ground using a hammer mill to pass a 0.5 mm screen and then analyzed for Ca, P, and Ti using inductively coupled plasma-optical emission spectrometry (**ICP-OES**, 5100 Dual View, Agilent, Santa Clara, CA) after sulfuric acid mineralization (based on method 985.01; AOAC International, 2006). Calcium, P, and Ti were then used to determine AID and ATTR using the Ti marker ratios in the diet and digesta or excreta (Osunbami and Adeola, 2022a). Phytate (InsP6) and the lower phytate esters (InsP5-2) in diet and digesta were analyzed by high-performance ion chromatography (**HPIC**) according to the method of Chen and Li (2003). Phytase activity in the diets were analyzed as previously described (Engelen et al., 1994).

### Experiment 2

#### Animals and Experimental Diets

Male Ross 308 chicks were obtained on d 0 posthatching and were subjected to the same housing and management practice described for Exp. 1. A nutrient-adequate common starter diet was fed from d 0 to 18 posthatching. On d 19, birds (432) were assigned to 8 experimental diets using a randomized complete block design replicated 9 times with 6 birds per replicate. Four diets were formulated to contain graded concentrations of SID Ca at 5.2, 4.2, 3.2, or 2.2 g/kg and fed to d 21 (Table 1). Nonphytate P in all diets was formulated at 2.2 g/kg. Phytase was added to the lowest SID Ca diets at 350, 700, 1,400, or 2,800 FYT/kg. The phytase was a novel phytase as described in Exp. 1 (HiPhorius, DSM Nutritional Products, Kaiseraugst, Switzerland). All 8 diets were formulated to be isoenergetic and isonitrogenous at metabolizable energy and crude protein of 3,000 kcal/kg and 230 g/kg. All diets contained 1.0 g/kg titanium dioxide as an inert marker for the determination of AID and ATTR of Ca and P.

#### Data Collection and Analysis

On d 19 and 21 posthatching, all birds were weighed and FI from d 19 to 21 was recorded based on the ad libitum feed consumption of 6 birds per cage. On d 21, all birds were euthanized by cervical dislocation. Digesta and excreta samples were collected, dried, and ground, and diets, digesta, and excreta were analyzed for Ca, P, and Ti as described in Exp. 1. Phytase activity in the diets were analyzed as previously described (Engelen et al., 1994).

### Experiment 3

#### Animals and Experimental Diets

Male Cobb 500 chicks were individually tagged for identification purposes on d 0 and reared in electrically heated battery cages (Alternative Design Manufacturing and Supply, Siloam Springs, AR) with a temperature of 35°C for d 0 to 7, and subsequently adjusted as described by Osunbami and Adeola (2022b). Light was provided 23 h per day throughout the study. Feed and water, analyzed to contain nondetectable concentration of Ca, were offered for ad libitum consumption. The water supply to the battery cages was passed through a water softening system. A standard starter diet formulated to meet or exceed the recommended nutrient according to NRC (1994) was fed from d 0 to 7 posthatching. On d 7, birds (1,152) were assigned to 8 experimental diets using a randomized complete block design replicated 12 times with 12 birds per replicate. The negative control (**NC**) diet was formulated to contain 5.1 g/kg of each of total Ca and total P. Phytase was added to the NC at 500, 1,000, 2,000, or 4,000 FYT/kg, and 3 graded levels of limestone at Ca concentrations of 1.3, 2.6, or 3.9 g Ca/kg were added to the NC for a total of 8 diets. All 8 diets were formulated to be isoenergetic and isonitrogenous at the respective metabolizable energy and crude protein of 3,048 kcal/kg and 230 g/kg (Table 2).

**Table 2:** Ingredient and calculated composition of the experimental diets, as-fed (Exp. 3).

Added Ca from limestone, g/kg:	3.9	2.6	1.3	0.0				
Supplemental phytase, FYT/kg:	0	0	0	0	500	1,000	2,000	4,000
Ingredients								
Corn	542.6	542.6	542.6	542.3	532.7	522.7	502.7	462.7
Soybean meal	373.0	373	373	373	373	373	373	373
Soybean oil	20.0	20	20	20	20	20	20	20
Salt	2.8	2.8	2.8	2.8	2.8	2.8	2.8	2.8
Limestone^1^	20.7	16.9	13.1	9.3	9.3	9.3	9.3	9.3
Monocalcium phosphate^2^	5.9	5.9	5.9	5.9	5.9	5.9	5.9	5.9
Celite	–	3.9	7.6	11.3	11.3	11.3	11.3	11.3
Sodium bicarbonate	2.0	2	2	2	2	2	2	2
Lysine HCl	1.4	1.4	1.4	1.4	1.4	1.4	1.4	1.4
DL-Methionine	2.9	2.9	2.9	2.9	2.9	2.9	2.9	2.9
Threonine	0.7	0.7	0.7	0.7	0.7	0.7	0.7	0.7
Vitamin and mineral premix^3^	3.0	3	3	3	3	3	3	3
Phytase premix^4^	–	–	–	–	10	20	40	80
Titanium dioxide^5^	25	25	25	25	25	25	25	25
Calculated nutrients, g/kg								
ME, kcal/kg	3,048	3,048	3,048	3,048	3,048	3,048	3,048	3,048
Crude protein	230	230	230	230	230	230	230	230
Ca	9.0	7.7	6.4	5.1	5.1	5.1	5.1	5.1
P	5.1	5.1	5.1	5.1	5.1	5.1	5.1	5.1

1 The limestone used in the study had a GMD of 870 um, a total Ca of 34.0% and an in vitro solubility (Kim et al., 2019) of 68.61% and 85.36% at 15 and 30 min, respectively. The estimated digestibility coefficient using the glycine buffer equation without phytase from Kim et al. (2019) was 0.651.

2 Contained 14.3% Ca and 21% P.

3 Supplied the following per kg diet: vitamin A, 5,145 IU; vitamin D3, 2,580 IU; vitamin E, 17.15 IU; menadione, 4.38 mg; riboflavin, 5.49 mg; D-pantothenic acid, 11.0 mg; niacin, 44.1 mg; choline chloride, 771 mg; vitamin B12, 0.01 mg; biotin, 0.06 mg; thiamine mononitrate, 2.20 mg; folic acid, 0.99 mg; pyridoxine hydrochloride, 3.30 mg; I, 1.11 mg; Mn, 107 mg; Cu, 4.44 mg; Fe, 73.5 mg; Zn, 179 mg; and Se, 0.43 mg.

4 Prepared with ground corn as 1 g HiPhorius 10 (10,000 FYT/g) added to 199 g corn to contain 50 FYT/g premix. Adding 10 g of premix per kg of diet gives 500 FYT phytase, 20 g of premix per kg of diet gives 1,000 FYT, 40 g of premix per kg of diet gives 2,000 FYT, and 80g of premix per kg of diet gives 4,000 FYT.

5 Prepared as 1 g titanium dioxide added to 4 g corn.

#### Data Collection and Analysis

On d 10 posthatching, all birds were weighed and FI from d 7 to 10 was recorded based on the ad libitum feed consumption of 12 birds per cage. Six of the 12 birds per cage were randomly selected and euthanized with CO_2_ asphyxiation, after which the left drumstick was excised. Similarly, on d 21, the remaining 6 birds in each cage were weighed and euthanized for calculation of body weight gain (**BWG**) and left drumstick collection as described above. Feed intake from d 10 to 21 was recorded based on the ad libitum feed consumption of the 6 birds per cage. The drumsticks were excarnated by autoclaving at 100°C for 15 min and the resulting tibias were lipid-extracted with ether and ashed for 16 h at 600°C in a muffle furnace to determine bone ash.

### Statistical Analyses

Data from Exp. 1 and 2 were subjected to a 1-way analysis of variance using JMP Pro v. 16.0 (SAS Institute, Cary NC). Cage served as the experimental unit. Prior to statistical analyses, the distribution platform was used to verify normality. Outliers, determined as 3 times the root mean square error plus or minus the mean of the response, were removed from the statistical analysis. Growth performance, tibia ash, AID, ATTR, and the calculated apparent ileal digested or apparent retained Ca or P were analyzed using the fit model platform. For all parameters, the statistical model included diet and split by day. Phytate, phytate esters, and myo-inositol data (Exp. 1) were transformed prior to statistical analyses and the untransformed data presented. If diet effects were significant, the impact of SID Ca concentration was evaluated using linear and quadratic contrasts, and the impact of phytase dose (including 0 FYT/kg) was evaluated using nonorthogonal linear and quadratic contrasts. The fit curve platform, analyzed the dose of phytase, analyzed total Ca, and measured apparent ileal digested Ca was used to estimate the digestible Ca and total Ca equivalency for phytase from 0 to 3,000 FYT/kg. The Ca equivalency was estimated using the direct method as described for P by Dersjant-Li et al. (2019) and the indirect method as described for P by Adedokun et al. (2004) or Adeola (2010).

The growth performance and tibia data from Exp. 3 were subjected to analysis of variance using SAS 9.4 (SAS Institute) with a cage as the experimental unit. The impact of graded Ca concentration was evaluated using linear and quadratic contrasts, and the impact of phytase dose (including 0 FYT/kg) was evaluated using nonorthogonal linear and quadratic contrasts. The IML procedure of SAS 9.4 was used to generate contrast coefficients for the unequally spaced graded level of phytase. Statistical significance was set at *P* < 0.05.

## RESULTS

### Experiment 1

#### Experimental Diets

The phytase activity recovered in the experimental diets was approximately 82 to 124% of the expected values (Table 3). Xylanase recoveries were lower than expected and protease was approximately 20 to 60% greater than expected (data not shown). The analyzed total Ca and total P were within the expected values. In general, graded concentrations of total Ca were achieved in the experimental diets (Table 3).

**Table 3:** Recovered enzyme activity and analyzed the nutrient content of the experimental diets on an as fed basis.^1^

		Experiment 1 (d 8 to 10)			Experiment 2 (d 19 to 21)			Experiment 3 (d 7 to 21)				
SID Ca,	Expected phytase,	Recovered phytase,	Total Ca,	Total P,	Recovered phytase,	Total Ca,	Total P,	Formulated total Ca,	Expected phytase,	Recovered phytase,	Total Ca,	Total P,
g/kg	g/kg	FYT/kg	g/kg	g/kg	FYT/kg	g/kg	g/kg	g/kg	FYT/kg	FYT/kg	g/kg	g/kg
5.2	0	–	8.5	5.4	–	8.9	4.8	9.0	0	–	9.2	5.2
4.2	0	–	8.2	5.0	–	7.3	4.9	7.7	0	–	7.6	5.1
3.2	0	–	5.7	4.8	–	5.7	5.0	6.4	0	–	6.6	5.2
2.2	0	–	4.2	4.9	–	4.1	5.0	5.1	0	–	5.3	5.1
	350	319			297				500	1,520		
	700	575			718				1,000	2,216		
	1,400	1,522			1,430				2,000	4,006		
	2,800	2,828			2,948				4,000	7,661		
		Experiment 1 (d 20 to 24)										
4.6	0	–	8.2	4.1								
3.6	0	–	5.9	4.1								
2.6	0	–	4.4	4.4								
1.6	0	–	2.7	4.3								
	350	346										
	700	869										
	1,400	1,322										
	2,800	2,519										

1 Diets were analyzed in duplicate.

#### Growth Performance and Tibia Ash

No birds were culled or died during the experimental periods. The mean BW on d 8 and 20 was 155 ± 1.3 g and 830 ± 40 g, respectively. There was no effect of diet on BW on d 8, 10, 20, or 24. Growth performance, including FI, BWG, or FCR were not influenced by diet from d 8 to 10 or d 20 to 24 (Table 4). Tibia ash percent or weight were not influenced by diet (d 24, Table 4).

**Table 4:** Growth performance of broilers fed graded concentrations of standardized ileal digestible (SID) calcium or phytase from d 8 to 10 or 20 to 24^1^ (Exp. 1) or d 19 to 21 posthatching^2^ (Exp. 2)

SID Ca, g/kg	Phytase, FYT/kg	Experiment 1 (d 8 to 10)			Experiment 1 (d 20 to 24)					Experiment 2 (d 19 to 21)		
		Feed intake,	BW gain,	FCR	Feed intake,	BW gain,	FCR	Tibia ash,	Tibia ash,	Feed intake,	BW gain,	FCR
		g	g	g:g	g	g	g:g	g/kg	mg/bone	g	g	g:g
5.2	0	66	62	1.109	286	156	1.927	417	449	312	187	1.639
4.2	0	76	61	1.247	341	189	1.849	415	455	313	210	1.494
3.2	0	67	64	1.033	343	180	1.970	404	456	305	227	1.348
2.2	0	75	65	1.142	315	163	1.963	407	446	318	235	1.358
	350	72	64	1.142	335	179	1.909	406	444	322	244	1.321
	700	74	66	1.126	307	150	2.211	413	459	328	255	1.293
	1,400	72	64	1.114	353	195	1.815	404	449	318	244	1.303
	2,800	78	66	1.174	329	165	2.082	401	480	313	241	1.302
SEM		3	2	0.052	15	13	0.153	7	26	9	6	0.036
*P*-value												
Diet		0.203	0.191	0.258	0.065	0.178	0.675	0.708	0.985	0.787	<0.001	<0.001
Linear SID Ca		–	–	–	–	–	–	–	–	–	<0.001	<0.001
Quadratic SID Ca		–	–	–	–	–	–	–	–	–	0.238	0.038
Linear phytase dose^3^		–	–	–	–	–	–	–	–	–	0.537	0.258
Quadratic phytase dose^3^		–	–	–	–	–	–	–	–	–	0.054	0.417

1 Data are least-square means of 11 (d 8 to 10) or 5 (d 20 to 24) birds per pen and 8 replicate pens per treatment.

2 Data are the least-square means of 6 birds per pen and 9 replicate pens per treatment.

3 Nonorthogonal contrasts were conducted including all doses of phytase (0 to 2,800 FYT/kg).

#### Apparent Nutrient Utilization and Calculated Digested Nutrients

Apparent ileal digestibility and ATTR of Ca linearly increased (*P <* 0.05) on d 10 and tended to linearly (*P <* 0.10) decrease on d 24 as the SID Ca level in the diet decreased (Table 5). Apparent ileal digested and retained Ca quadratically decreased (*P <* 0.05) at both d 10 and d 24 as the SID Ca concentration decreased in the diets. Phytase supplementation quadratically (*P <*0.05) increased the AID, ATTR, ileal digested, and retained Ca on d 10 and linearly (*P <* 0.05) increased the AID, ATTR, ileal digested, and retained Ca on d 24.

**Table 5:** Apparent ileal digestibility and total tract retention of calcium in broilers fed graded concentrations of standardized ileal digestible (SID) calcium or phytase from d 8 to 10 or d 20 to 24 posthatching^1^ (Exp. 1).

SID Ca, g/kg (starter/grower)	Phytase, FYT/kg	Apparent ileal digestibility of Ca, g/kg		Apparent ileal digested Ca, g/kg		Apparent total tract retention of Ca, g/kg		Apparent retained Ca, g/kg	
		d 8 to 10	d 20 to 24	d 8 to 10	d 20 to 24	d 8 to 10	d 20 to 24	d 8 to 10	d 20 to 24
5.2/4.6	0	497.1	548.1	4.23	4.49	318.1	442.6	2.60	3.63
4.2/3.6	0	571.3	504.8	4.46	2.96	404.0	357.2	3.31	2.11
3.2/2.6	0	556.6	485.6	3.17	2.14	393.1	379.5	2.25	1.67
2.2/1.6	0	574.4	496.1	2.41	1.39	461.9	284.7	1.94	0.80
2.2/1.6	350	682.2	530.8	2.87	1.49	566.4	350.0	2.38	0.98
	700	688.6	580.4	2.89	1.62	556.2	370.5	2.34	1.02
	1,400	680.2	554.6	2.86	1.55	566.0	436.3	2.38	1.22
	2,800	690.6	617.4	2.92	1.81	572.5	483.2	2.41	1.35
SEM		17.1	21.9	0.09	0.09	19.7	24.3	0.09	0.10
*P*-value									
Diet		<0.001	0.002	<0.001	<0.001	<0.001	<0.001	<0.001	<0.001
Linear SID Ca		0.007	0.082	<0.001	<0.001	<0.001	<0.001	<0.001	<0.001
Quadratic SID Ca		0.106	0.236	<0.001	0.001	0.666	0.846	<0.001	0.002
Linear phytase dose^2^		0.001	0.005	0.002	0.003	0.001	<0.001	0.002	0.001
Quadratic phytase dose^2^		0.002	0.820	0.018	0.741	0.019	0.926	0.032	0.897

1 Data are least-square means of 6 birds per pen from d 8 to 10 or 5 birds per pen from d 20 to 24 and 8 replicate pens per treatment.

2 Nonorthogonal contrasts were conducted including all doses of phytase (0 to 2,800 FYT/kg).

The AID and apparent ileal digested P quadratically (*P <* 0.05) increased as the SID Ca concentration of the diet decreased on d 10; but only linearly on d 24 posthatching (Table 6). The ATTR of P increased quadratically (*P <* 0.05) on d 10 and decreased quadratically (*P* < 0.05) on d 24 as the SID level in the diet decreased. Similarly, apparent retained P quadratically (*P <* 0.05) decreased as SID Ca in the diet decreased on d 10 and d 24. Phytase supplementation linearly and quadratically (*P <* 0.01) increased the AID, apparent ileal digested, and the ATTR and retained P on d 10, with linear (*P <* 0.01) and quadratically tendencies (*P <* 0.10) on d 24.

**Table 6:** Apparent ileal digestibility and total tract retention of phosphorus in broilers fed graded concentrations of standardized ileal digestible (SID) calcium or phytase from d 8 to 10 or d 20 to 24 posthatching^1^ (Exp. 1).

SID Ca, g/kg (starter/grower)	Phytase, FYT/kg	Apparent ileal digestibility of P, g/kg		Apparent ileal digested P, g/kg		Apparent total tract retention of P, g/kg		Apparent retained P, g/kg	
		d 8 to 10	d 20 to 24	d 8 to 10	d 20 to 24	d 8 to 10	d 20 to 24	d 8 to 10	d 20 to 24
5.2/4.6	0	495.2	536.0	2.68	2.20	452.4	470.1	2.45	1.93
4.2/3.6	0	481.2	551.0	2.41	2.26	453.8	459.8	2.27	1.88
3.2/2.6	0	541.5	592.0	2.60	2.61	457.1	476.2	2.19	2.10
2.2/1.6	0	625.5	644.3	3.06	2.71	497.7	385.9	2.44	1.62
2.2/1.6	350	726.2	720.4	3.56	3.03	523.7	401.9	2.57	1.69
	700	791.4	760.6	3.88	3.19	533.8	378.8	2.62	1.59
	1,400	825.4	780.4	4.05	3.28	544.0	403.1	2.67	1.69
	2,800	832.4	829.0	4.08	3.48	565.2	417.3	2.77	1.75
SEM		9.5	11.3	0.05	0.05	8.3	12.2	0.04	0.05
*P*-value									
Diet		<0.001	<0.001	<0.001	<0.001	<0.001	<0.001	<0.001	<0.001
Linear SID Ca		<0.001	<0.001	<0.001	<0.001	0.001	0.001	0.591	0.003
Quadratic SID Ca		<0.001	0.106	<0.001	0.695	0.021	0.002	<0.001	0.001
Linear phytase dose^2^		<0.001	<0.001	<0.001	<0.001	<0.001	0.102	<0.001	0.100
Quadratic phytase dose^2^		<0.001	0.082	<0.001	0.084	0.757	0.349	0.753	0.358

1 Data are least-square means of 6 birds per pen from d 8 to 10 or 5 birds per pen from d 20 to 24 and 8 replicate pens per treatment.

2 Nonorthogonal contrasts were conducted including all doses of phytase (0 to 2,800 FYT/kg).

#### Phytate (InsP6) and Lower Phytate (InsP5-2) Concentration in the Ileum

Reducing dietary SID Ca linearly decreased (*P <* 0.01) the concentration of InsP6 or InsP5 on both d 10 and 24 (Table 7). Furthermore, increasing the dose of phytase quadratically (*P <* 0.01) decreased the concentration of InsP6 or InsP5 on d 10. The concentration of InsP4 or InsP3 was below the limits of detection in all diets and there was no effect of diet on InsP2 regardless of age.

**Table 7:** Phytate (InsP6) and lower phytate ester (InsP5-2) concentration in the ileum of broilers fed graded concentrations of standardized ileal digestible (SID) calcium or phytase from d 8 to 10 or d 20 to 24 posthatching^1^ (Exp. 1).

SID Ca, g/kg (starter/grower)	Phytase, FYT/kg	InsP6, µmol/mg		InsP5, µmol/mg		InsP4, µmol/mg		InsP3, µmol/mg		InsP2, µmol/mg	
		d 8 to 10	d 20 to 24	d 8 to 10	d 20 to 24	d 8 to 10	d 20 to 24	d 8 to 10	d 20 to 24	d 8 to 10	d 20 to 24
5.2/4.6	0	34.37	28.74	4.22	2.83	BLD	BLD	BLD	BLD	36.61	38.03
4.2/3.6	0	30.00	27.26	3.33	2.43	BLD	BLD	BLD	BLD	36.55	35.89
3.2/2.6	0	25.50	23.44	2.96	1.99	0.54	BLD	BLD	BLD	36.08	35.07
2.2/1.6	0	21.41	20.43	3.04	1.28	BLD	BLD	BLD	BLD	35.59	34.17
2.2/1.6	350	7.21	11.29	0.69	0.94	1.53	BLD	BLD	BLD	35.17	35.42
	700	3.68	9.59	0.65	0.31	0.44	BLD	BLD	BLD	35.51	36.90
	1,400	2.80	6.39	0.01	BLD	BLD	BLD	BLD	BLD	39.01	33.36
	2,800	1.61	4.88	BLD	0.29	BLD	BLD	BLD	BLD	34.24	36.83
SEM		1.90	1.79	0.34	0.34	–	–	–	–	1.23	1.42
*P*-value											
Diet		<0.001	<0.001	<0.001	<0.001	–	–	–	–	0.322	0.315
Linear SID Ca		0.002	0.005	0.006	0.005	–	–	–	–	–	–
Quadratic SID Ca		0.933	0.745	0.204	0.621	–	–	–	–	–	–
Linear phytase dose^2^		<0.001	<0.001	<0.001	0.008	–	–	–	–	–	–
Quadratic phytase dose^2^		0.004	0.144	0.003	0.205	–	–	–	–	–	–

1 Nonnormally distributed data (InsP6, InsP5, and InsP4) were transformed prior to analyses and are presented as the least squares means of 6 birds per pen from d 8 to 10 and 8 replicate pens per treatment.

2 Nonorthogonal contrasts were conducted including all doses of phytase (0 to 2,800 FYT/kg). Abbreviation: BLD, below limits of detection.

### Experiment 2

#### Experimental Diets

Phytase recoveries in the diets were between 85 to 105% of the expected values (Table 3). Recoveries of added dietary xylanase (66 to 117% of the expected values) and protease (much closer to the expected value) were highly variable (data not shown). The analyzed total Ca and total P were within expected values (Table 3).

#### Growth Performance

No birds were culled or died during the experimental period (d 19 to 21 posthatching). There was no impact of diet on FI from d 19 to 21 or final BW (d 21; Table 4). Body weight gain increased (linear, *P <* 0.01) and FCR decreased (quadratic, *P <* 0.05) as SID Ca content in the diet decreased from 5.2 g/kg to 2.2 g/kg (Table 4). Dietary phytase supplementation had no effect on any growth performance response criterion during the d 19 to 21 experimental period. Tibias were not collected due to no effect of the experimental diets on tibia ash in Exp. 1.

#### Apparent Nutrient Utilization and Calculated Digested Nutrients

The AID and ATTR of Ca increased (linear, *P <* 0.05) as the SID concentration in the diet decreased from 5.2 to 2.2 g/kg (Table 8). Apparent ileal digested and apparent retained Ca linearly decreased (*P <* 0.01) as the SID Ca level in the diet decreased. Increasing phytase dose linearly increased (*P <* 0.01) the AID, apparent ileal digested, ATTR, and retained Ca. The AID and apparent ileal digested P linearly and quadratically increased (*P <* 0.01) and the ATTR and apparent retained P linearly increased (*P <* 0.01) as the SID Ca content in the diet decreased from 5.2 to 2.2 g/kg (Table 8). Phytase supplementation linearly and quadratically (*P <* 0.01) increased the AID and apparent ileal digested P as the phytase dose increased. In addition, increasing phytase dose linearly increased (*P <* 0.01) ATTR and retained P.

**Table 8:** Apparent utilization and retention of Ca and P in broilers fed graded concentrations of standardized ileal digestible (SID) calcium or phytase from d 19 to 21 posthatching^1^ (Exp. 2).

SID Ca, g/kg	Phytase, FYT/kg	Apparent ileal digestibility of Ca,	Apparent ileal digested Ca,	Apparent total tract retention of Ca,	Apparent retained Ca,	Apparent ileal digestibility of P,	Apparent ileal digested P,	Apparent total tract retention of P,	Apparent retained P,
		g/kg	g/kg	g/kg	g/kg	g/kg	g/kg	g/kg	g/kg
5.2	0	435.1	3.75	203.8	1.82	341.8	1.63	315.7	1.51
4.2	0	447.8	3.28	227.4	1.66	386.0	1.89	372.7	1.82
3.2	0	440.9	2.51	284.0	1.61	414.6	2.07	415.3	2.07
2.2	0	495.8	2.02	374.8	1.53	528.7	2.64	465.3	2.32
2.2	350	554.3	2.28	453.3	1.86	665.5	3.32	488.1	2.44
	700	534.3	2.17	465.5	1.88	720.2	3.61	500.9	2.51
	1,400	563.2	2.34	489.9	2.04	751.4	3.77	510.3	2.56
	2,800	606.3	2.54	526.1	2.20	794.4	3.97	517.4	2.59
SEM		15.1	0.07	19.7	0.10	9.3	0.05	11.1	0.06
*P*-value									
Diet		<0.001	<0.001	<0.001	0.001	<0.001	<0.001	<0.001	<0.001
Linear SID Ca		0.012	<0.001	<0.001	0.034	<0.001	<0.001	<0.001	<0.001
Quadratic SID Ca		0.168	0.917	0.094	0.716	0.005	0.001	0.990	0.606
Linear phytase dose^2^		<0.001	<0.001	<0.001	<0.001	<0.001	<0.001	0.001	0.005
Quadratic phytase dose^2^		0.749	0.576	0.331	0.540	<0.001	<0.001	0.406	0.340

1 Data are the least square means of 6 birds per pen and 9 replicate pens per treatment.

2 Nonorthogonal contrasts were conducted including all doses of phytase (0 to 2,800 FYT/kg).

### Experiment 3

#### Experimental Diets

Analyzed Ca and P content in the diets were within the acceptable range (Table 3). Phytase activity was largely out of the formulated range as diets calculated to contain 500, 1,000, 2,000, or 4,000 FYT/kg returned analyzed values of 1,520, 2,216, 4,006, or 7,661 FYT/kg, respectively (Table 3).

#### Growth Performance and Tibia Ash

Mean BW on d 7, 10, and 21 were 159 ± 16 g, 254 ± 24 g, and 861 ± 108 g, respectively. Growth performance, including FI, BWG, and FCR were influenced (*P <* 0.01) by diet from d 7 to 10 or d 7 to 21 (Table 9). At 10 d of age, decreasing the levels of Ca linearly increased (*P <* 0.01) BWG and FI, whereas increasing phytase supplementation linearly increased (*P <* 0.01) BWG and decreased FCR during d 7 to 10 or d 7 to 21 (Table 9). Also, increasing phytase supplementation quadratically decreased (*P <* 0.05) FI during d 7 to 10 and linearly (*P <* 0.01) during d 7 to 21. Patterns similar to the d 10 results were observed in the older (d 21) birds as the decreased dietary SID Ca concentration resulted in a linear improvement (*P <* 0.01) in BWG and FCR (Table 9). In addition, BWG increased and FCR decreased linearly and quadratically (*P <* 0.05) with phytase supplementation. Also, decreasing SID Ca concentration linearly increased (*P <* 0.05) FI, whereas increasing phytase supplementation linearly increased (*P <* 0.05) FI. Tibia dry matter weight and ash percent were influenced by diet from d 7 to 10 or d 7 to 21, whereas ash weight was neither influenced from d 7 to 10 nor d 7 to 21 (Table 10). Dietary SID Ca affected tibia ash percent on d 21, perhaps due to the longer (14 d) feeding period. Decreasing the levels of Ca linearly decreased (*P <* 0.05) tibia ash percent on d 21 posthatching. On d 10 and 21 posthatching, increasing the concentration of dietary phytase increased (linear and quadratic, *P <* 0.05) tibia dry matter weight, whereas tibia ash percent was decreased (linear and quadratic, *P <* 0.05).

**Table 9:** Growth performance in broilers fed graded concentrations of calcium from limestone or phytase from d 7 to 10 or d 7 to 21 posthatching^1^ (Exp. 3).

		Feed intake, g		Body weight gain, g		mFCR, g:g	
Total Ca, g/kg	Phytase, FYT/kg	d 7 to 10	d 7 to 21	d 7 to 10	d 7 to 21	d 7 to 10	d 7 to 21
9.0	0	106	661	81	466	1.318	1.419
7.7	0	115	731	89	529	1.296	1.385
6.4	0	113	764	88	562	1.279	1.361
5.1	0	122	806	96	590	1.266	1.368
5.1	500	126	830	99	639	1.281	1.300
	1,000	127	856	99	667	1.287	1.284
	2,000	130	860	104	676	1.254	1.273
	4,000	119	886	102	705	1.156	1.261
SEM		2.6	17.0	1.8	14.0	0.028	0.013
*P*-value							
Diet		<0.001	<0.001	<0.001	<0.001	0.003	<0.001
Linear Ca		0.002	<0.001	<0.001	<0.001	0.152	0.003
Quadratic Ca		0.847	0.417	0.875	0.220	0.860	0.106
Linear phytase dose^2^		0.183	0.001	0.006	<0.001	0.001	<0.001
Quadratic phytase dose^2^		0.002	0.292	0.055	0.020	0.107	0.001

1 Data are least-square means of 12 birds per pen from d 7 to 10 or 6 birds per pen from d 7 to 21 and 12 replicate pens per treatment.

2 Nonorthogonal contrasts were conducted, including all doses of phytase (0 to 4,000 FYT/kg).

**Table 10:** Bone characteristics in broilers fed graded concentrations of calcium from limestone or phytase from d 7 to 10 or d 7 to 21 posthatching^1^ (Exp. 3).

		Tibia dry matter weight, g		Ash weight, g		Ash, %	
Total Ca, g/kg	Phytase, FYT/kg	d 7 to 10	d 7 to 21	d 7 to 10	d 7 to 21	d 7 to 10	d 7 to 21
9.0	0	2.07	8.86	1.24	5.58	59.9	65.0
7.7	0	2.05	9.32	1.22	5.76	59.6	61.7
6.4	0	2.00	9.39	1.20	5.53	59.9	58.3
5.1	0	2.08	9.54	1.22	5.36	58.4	56.1
5.1	500	2.30	10.42	1.23	5.25	53.6	50.4
	1,000	2.31	11.60	1.24	5.73	53.8	49.4
	2,000	2.40	11.56	1.24	5.76	51.7	50.0
	4,000	2.26	11.88	1.14	5.83	49.7	49.1
SEM		0.04	0.28	0.04	0.19	1.1	1.1
*P*-value							
Diet		<0.001	<0.001	0.546	0.294	<0.001	<0.001
Linear Ca		0.973	0.095	0.575	0.307	0.392	<0.001
Quadratic Ca		0.281	0.578	0.654	0.358	0.596	0.618
Linear phytase dose^2^		0.054	<0.001	0.082	0.032	<0.001	0.001
Quadratic phytase dose^2^		<0.001	0.001	0.137	0.297	0.047	0.004

1 Data are the least-square means of 6 birds per pen from d 7 to 10 or d 7 to 21 and 12 replicate cages per treatment.

2 Nonorthogonal contrasts were conducted, including all doses of phytase (0 to 4,000 FYT/kg).

#### Calcium Equivalence of Phytase

Due to the lack of diet effects on growth performance or tibia ash, it was not possible to estimate the Ca equivalence of phytase for growth performance or tibia ash using the indirect method and graded levels of SID Ca in Exp. 1. However, apparent ileal digested Ca was influenced by diet; with a good agreement (r = 0.84, *P <* 0.0001 or r = 0.96, *P <* 0.0001) between the formulated SID Ca and the measured AID Ca concentrations on d 8 or d 24, respectively, and a quadratic or linear effect of phytase. Therefore, the digestible and total Ca equivalence of phytase was estimated using analyzed phytase doses from 500 to 2,500 FYT/kg using the direct or indirect method (Table 11). In general, 500 to 2,500 FYT/kg resulted in a digestible or total Ca equivalence of 0.99 or 2.15 g/kg, respectively, from d 8 to 10. However, from d 20 to 24, the Ca equivalence was much lower at 0.07 to 0.39 g/kg digestible Ca or 0.13 to 0.67 g/kg total Ca from 500 to 2,500 FYT/kg. There was good agreement between direct and indirect method for the estimated digestible Ca equivalence at each dose of phytase.

**Table 11:** Estimated digestible calcium equivalency of graded levels of phytase in broiler chickens.

	Experiment 1						Experiment 2		
Method	Direct method^1^		Indirect method^2^				Direct method^1^		Indirect method^2^
Response	Digestible Ca, g/kg		Digestible Ca, g/kg		Total Ca, g/kg		Digestible Ca, g/kg		Total Ca, g/kg
Age	d 8 to 10	d 20 to 24	d 8 to 10	d 20 to 24	d 8 to 10	d 20 to 24	d 19 to 21		
Model	Exponential	Linear	Exponential	Linear	Exponential	Linear	Linear	Linear	
R^2^	0.65	0.32	0.65	0.32	0.65	0.32	0.38	0.38	
AICc	−214	−194	−214	−194	−214	−194	−193	−193	
RMSE	0.01	0.02	0.01	0.02	0.01	0.02	0.02	0.02	
Phytase, FYT/kg									
500	0.99	0.08	0.99	0.07	2.15	0.13	0.07	0.08	0.20
750	0.99	0.12	0.99	0.11	2.15	0.20	0.11	0.12	0.30
1,000	0.99	0.15	0.99	0.15	2.15	0.27	0.15	0.16	0.40
1,500	0.99	0.23	0.99	0.23	2.15	0.40	0.22	0.23	0.60
2,000	0.99	0.31	0.99	0.30	2.15	0.53	0.30	0.31	0.81
2,500	0.99	0.39	0.99	0.38	2.15	0.67	0.37	0.38	1.01
3,000	–	–	–	–	–	–	0.45	0.45	1.21

1 The direct method estimated the digestible Ca equivalency using the apparent ileal digested Ca of the lowest Ca diet without and with phytase.

2 The indirect method employed the graded levels of measured apparent ileal digested Ca and analyzed total Ca with graded doses of phytase. AICc = Akaike Information Criterion corrected for a small sample size. Abbreviation: RMSE, root mean square error.

As in Exp. 1, the lack of phytase effects on growth performance did not allow estimating the Ca equivalence of phytase using the indirect method with graded levels of SID Ca in Exp. 2. However, apparent ileal digested Ca was influenced by diet; with a good agreement (r = 0.94, *P* < 0.0001) between the formulated SID Ca and the measured AID Ca concentrations and quadratic or linear effects of phytase. Therefore, the digestible Ca equivalence from phytase was estimated to range from 0.07 to 0.45 g/kg and the total Ca equivalence was estimated to range from 0.20 to 1.21 g/kg as the analyzed phytase dose increased from 500 to 3,000 FYT/kg (Table 11). There was good agreement between direct and indirect method for the estimated digestible Ca equivalence at each dose of phytase.

## DISCUSSION

The indirect method has previously been successfully used to estimate the P equivalence of phytase (Adedokun et al., 2004; Adeola, 2010; Dersjant-Li et al., 2019). This method relies on the regression of BWG or tibia ash responses to graded dietary levels of inorganic P as well as responses to sequential increases in dietary phytase dose. In the current study, one objective was to use the indirect method and graded levels of limestone to estimate the Ca equivalence of phytase. However, the lack of an effect of dietary Ca and/or phytase supplementation on growth performance or tibia ash in Exp. 1, the lack of an effect of phytase in Exp. 2, or the nature of the dietary Ca and phytase responses in Exp. 3, meant it was not possible to estimate the Ca equivalence of phytase using the indirect method for performance or tibia ash as it had been developed for P equivalence. These results are unfortunate, but not entirely unexpected due to the short duration of the studies, the known imbalanced Ca to P ratios in the different diets, and the novel application of the test method. Gautier et al. (2017) reported linear reductions in BWG and tibia ash or strength as dietary Ca to nPP ratios increased from 1.33 to 5.33. Whereas Walk et al. (2021, 2022) reported significant quadratic improvements in tibia ash of broilers fed graded levels of dietary Ca in P-adequate diets. Similarly, David et al. (2021, 2022) reported significant effects of graded levels of both dietary Ca and dietary P on growth performance and tibia ash of broilers from hatch to d 24 of age.

The linear improvement in FI, BWG, or FCR as dietary Ca decreased in Exp. 2 and Exp. 3 (d 7 to 10) was most likely due to a narrowing of the Ca to P ratio in the experimental diets and this agrees with previous studies in broilers from hatch to d 21 (Gautier et al., 2017). Phytase supplementation linearly or quadratically increased BWG and decreased FCR at all time points in Exp. 3. However, the effect of phytase was greater than the impact of the graded levels of dietary Ca on growth performance and this eliminated any possibility of using the indirect method to estimate Ca equivalence of phytase for growth performance or tibia ash. As a follow-up to this study, perhaps maintaining a constant Ca to P ratio with the graded concentrations of dietary Ca or ensuring all diets are only marginally deficient in P or even adequate in P would mitigate the profound impact the Ca to P ratio had on the results from the current study, thereby allowing estimation of a Ca equivalence value for phytase from the indirect method and growth performance.

When the study diets were fed from d 7 to 21 in Exp. 3, reducing dietary Ca linearly reduced tibia ash and provided an inorganic Ca dose response to estimate Ca equivalence. However, phytase supplementation resulted in a lower tibia ash percent compared with all concentrations of dietary Ca without phytase. Again, this was unfortunate and eliminated any possibility of estimating a Ca equivalence of phytase for growth performance or tibia ash using an indirect method. The reduction of tibia ash with phytase supplementation was surprising but may be a result of the difference in weight of the birds and the tibias when fed phytase, which is not considered in tibia ash percent. For example, Li et al. (2015) found that tibia ash weight, instead of tibia ash percent, was a better response criterion to reflect bone mineralization because ash percent does not consider differences in bone length, weight, or thickness. In the current study, tibia dry matter weight and ash weight were not influenced by dietary Ca levels, but significantly increased with phytase supplementation, especially from d 7 to 21. Therefore, feeding the experimental diets for longer than 48 h and using tibia ash weight, tibia Ca or P content, tibia length, and bone-breaking strength may be better response variables to use when using the indirect method to estimate the Ca equivalence of a phytase.

The second method to determine the nutrient release values for phytase is the direct method. The direct method provides an estimate of the digested or retained nutrients released by graded concentrations of phytase, compared with a nonsupplemented, nutrient-deficient control diet (Dersjant-Li et al., 2019). To minimize the adaptation of the birds to the experimental diets and only measure the effect of phytase, it has been suggested to only feed the test diets for <48 h (Li et al., 2018; Babatunde et al., 2019b). Feeding the test diets for 48 h or longer in the current studies may have allowed the birds to adapt to the Ca and P deficiencies in the diets and this could result in under-estimations of the Ca or P equivalency of phytase (Babatunde et al., 2019a, 2019b). The adaptation of the birds to the diets may be reflected in the linear or quadratic increases in the AID or ATTR of Ca and P as dietary Ca was reduced in the diets. Phytase supplementation significantly increased the AID and ATTR of both Ca and P and this has been previously reported (Ravindran et al., 2006; Selle et al., 2009; Babatunde et al., 2019a, 2019b; Li et al., 2021). Future studies designed to estimate the digestible nutrient equivalence of phytase may need to consider feeding the test diets for <48 h to avoid potential adaptation of broilers to the diets. If using older birds (>20 d of age), perhaps an even shorter adaptation time on the test diets may suffice. For example, Walk et al. (2023) observed that the effect of 2 concentrations of dietary Ca on the AID of Ca was already apparent by 8 h on the test diets in 24-day-old broilers, whereas this was only apparent by 48 h in 10-day-old broilers. Perhaps a mature gastrointestinal tract in older birds allows for earlier adaptation to nutrient deficiencies in the test diets.

Regardless of the hypothesized adaptation of the birds to the experimental diets, there was good agreement between the formulated SID Ca and the measured apparent ileal digested Ca in Exp. 1 (d 10 and d 24) or Exp. 2 (r = 0.84, *P* < 0.0001; r = 0.96, *P* < 0.0001; r = 0.94, *P* < 0.0001, respectively). The linear or quadratic decrease in the apparent ileal digested Ca as the dietary Ca concentration in the diets decreased and the linear and quadratic effects of phytase, allowed for the estimation of the total and digestible Ca equivalence from phytase at the measured doses of 500 to 3,000 FYT/kg using the direct or indirect methods. From d 8 to 10 in Exp. 1, 500 to 2,500 FYT/kg of phytase resulted in a digestible or total Ca equivalence of 0.99 g/kg or 2.15 g/kg, respectively. However, from d 20 to 24, the Ca equivalence was much lower at 0.07 to 0.39 g/kg digestible Ca or 0.13 to 0.67 g/kg total Ca from 500 to 2,500 FYT/kg. In Exp. 2, the digestible Ca equivalence from phytase was estimated to range from 0.07 to 0.45 g/kg and the total Ca equivalence was estimated to range from 0.20 to 1.21 g/kg as phytase dose increased from 500 to 3,000 FYT/kg. Within each age and experiment, there was good agreement between the direct and indirect method for the estimated digestible Ca equivalence at each dose of phytase, whereas it was not possible to estimate the total Ca equivalence using the direct method.

To our knowledge, these are the first examples of experiments designed to estimate the Ca equivalence of phytase using indirect and direct methods. Unfortunately, due to the nature of the BWG and tibia ash responses to both dietary Ca and phytase, it was not possible to estimate the Ca equivalence using the indirect method and graded concentrations of dietary Ca. However, future studies designed to evaluate the Ca equivalence of phytase, using the indirect method, could try to maintain a constant Ca to P ratio in the test diets, vary the Ca to P levels in the diets with phytase, feed the test diets for longer than 48 h, ensure P is marginally deficient, and consider using tibia ash weight, tibia Ca or P, and bone breaking strength as response criteria. Tibia ash percent might not be truly reflective of the mineral concentration in the diet. In addition, when employing the direct method to estimate the Ca equivalence for phytase, the age of the broilers and length of time on the test diets appears to have a big impact on the AID or ATTR. Consider feeding the test diets for <48 h in young birds and perhaps only 24 h in older birds to prevent adaptation to the deficiencies of the diets and ensure the effects of phytase are not underestimated. Employing determined digestible Ca for both the indirect and direct method resulted in a good agreement in the digested Ca equivalence of phytase and these results may indicate the direct method is sufficient for estimating the Ca equivalence of phytase. However, when evaluating the nutrient release values for any phytase, it is important to consider the results from numerous studies, using various concentrations of phytase, phytate, and perhaps different levels of Ca, P, and Ca to P ratios.

## References

[bib0001] Adedokun S.A., Sands J.S., Adeola O. (2004). Determining the equivalent phosphorus released by an *Escherichia coli*-derived phytase in broiler chicks. Can. J. Anim. Sci..

[bib0002] Adeola O. (2010). Phosphorus equivalency value of an *Escherichia coli* phytase in the diets of White Pekin ducks. Poult. Sci..

[bib0003] AOAC (2006).

[bib0004] Aureli R., Ueberschlag Q., Klein F., Noel C., Guggenbuhl P. (2017). Use of near infrared reflectance spectroscopy to predict phytate phosphorus, total phosphorus, and crude protein of common poultry feed ingredients. Poult. Sci..

[bib0005] Babatunde O.O., Cowieson A.J., Wilson J., Adeola O. (2019). Influence of age and duration of feeding low-phosphorus diet on phytase efficacy in broiler chickens during the starter phase. Poult. Sci..

[bib0006] Babatunde O.O., Cowieson A.J., Wilson J.W., Adeola O. (2019). The impact of age and feed length on phytase efficacy during the starter phase of broiler chickens. Poult. Sci..

[bib0007] Chen Q.-C., Li B.W. (2003). Separation of phytic acid and other related inositol phosphates by high-performance ion chromatography and its application. J. Chromatography A..

[bib0008] David L.S., Abdollahi M.R., Bedford M.R., Ravindran V. (2021). Requirement of digestible calcium at different dietary concentrations of digestible phosphorus for broiler chickens. 1. Broiler starters (d 1 to 10 post-hatch). Poult. Sci..

[bib0009] David L.S., Abdollahi M.R., Bedford M.R., Ravindran V. (2022). Requirement of digestible calcium at different dietary concentrations of digestible phosphorus for broiler chickens. 2. Broiler growers (d 11 to 24 post-hatch). Poult. Sci..

[bib0010] Davin R., Kwakernaak C., Dersjant-Li Y. (2020). Effect of two commercial limestone sources with different solubility on the efficacy of two phytases in 0-21 d old broilers. J. Appl. Anim. Nutr..

[bib0011] Dersjant-Li Y., Hruby M., Evans C., Greiner R. (2019). A critical review of methods used to determine phosphorus and digestible amino acid matrices when using phytase in poultry and pig diets. J. Appl. Anim. Nutr..

[bib0012] Engelen A.J., van der Heeft F.C., Randsdorp P.H., Smit E.L.J. (1994). Simple and rapid determination of phytase activity. AOAC Int.

[bib0013] Gautier A.E., Walk C.L., Dilger R.N. (2017). Influence of dietary calcium concentrations and the calcium to non-phytate phosphorus ratio on growth performance, bone characteristics, and digestibility in broilers. Poult. Sci..

[bib32] Kim S.-W., Li W., Angel R., Plumstead P.W. (2019). Modification of a limestone solubility method and potential to correlate with in vivo limestone calcium digestibility. Poult. Sci..

[bib0014] Li W., Angel R., Kim S.-W., Jimenez-Moreno E., Proszkowiec-Weglarz M., Plumstead P.W. (2015). Impact of response criteria (tibia ash weight vs. percent) on phytase relative non phytate phosphorus equivalence. Poult. Sci..

[bib0015] Li W., Angel R., Kim S.-W., Jimenez-Moreno E., Proszkowiec-Weglarz M., Plumstead P.W. (2018). Impacts of age and calcium on phytase efficacy in broiler chickens. Anim. Feed Sci. Technol..

[bib0016] Li W., Angel R., Plumstead P.W., Enting H. (2021). Effects of limestone particle size, phytate, calcium source, and phytase on standardized ileal calcium and phosphorus digestibility in broilers. Poult. Sci..

[bib0017] Liu N., Ru Y.J., Li F.D., Cowieson A.J. (2008). Effect of diet containing phytate and phytase on the activity and messenger ribonucleic acid expression of carbohydrase and transporter in chickens. J. Anim. Sci..

[bib33] National Research Council (1994). Nutrient requirements of poultry.

[bib0018] Nelson T.S., McGillivray J.J., Shieh T.R., Wodninski R.J., Ware J.H. (1968). Effect of phytate on the calcium requirement of chicks. Poult. Sci..

[bib0019] Novotny M.V.Sommerfeld, Krieg J., Kuhn I., Huber K., Rodehutscord M. (2023). Mucosal phosphatase activity, phytate degradation, and mineral digestibility in 6-week-old turkeys and broilers at different dietary levels of phosphorus and phytase and comparison with 3-week-old animals. Poult. Sci..

[bib0020] Osunbami O.T., Adeola O. (2022). Energy value of hydrolyzed feather meal and flash-dried poultry protein for broiler chickens and pigs. J. Anim. Sci..

[bib0021] Osunbami O.T., Adeola O. (2022). Regression method-derived digestible and metabolizable energy concentrations of partially defatted black soldier fly larvae meal for broiler chickens and pigs. Livest. Sci..

[bib0022] Ravindran V., Morel P.C.H., Partridge G.G., Hruby M., Sands J.S. (2006). Influence of an Escherichia coli-derived phytase on nutrient utilization in broilers starters fed diets containing varying concentrations of phytic acid. Poult. Sci..

[bib0023] Selle P.H., Cowieson A.J., Ravindran V. (2009). Consequences of calcium interactions with phytate and phytase for poultry and pigs. Livestock Sci.

[bib0024] Sommerfeld V., Schollenberger M., Kuhn I., Rodehutscord M. (2018). Interactive effects of phosphorus, calcium, and phytase supplements on products of phytate degradation in the digestive tract of broiler chickens. Poult. Sci..

[bib0025] Tahir M., Shim M.Y., Ward N.E., Smith C., Foster E., Guney A.C., Pesti G.M. (2012). Phytate and other nutrient components of feed ingredients for poultry. Poult. Sci..

[bib0026] Walk C.L., Bedford M.R., Santos T.T., Paiva D., Bradley J.R., Wladecki H., Honaker C., McElroy A.P. (2013). Extra-phosphoric effects of superdoses of a novel microbial phytase. Poult. Sci..

[bib0027] Walk C.L., Wang Z., Wang S., Wu J., Sorbara J.O.B., Zhang J. (2021). Determination of the standardized ileal digestible calcium requirement of male Arbor Acres Plus broilers from hatch to day 10 post-hatch. Poult. Sci..

[bib0028] Walk C.L., Wang Z., Wang S., Sorbara J.O.B., Zhang J. (2022). Determination of the standardized ileal digestible calcium requirement of male Arbor Acres Plus broilers from day 11 to 24 post-hatch. Poult. Sci..

[bib0029] Walk C.L., Veluri S., Olukosi O.A. (2023). Ileal mineral digestibility and expression of nutrient transporter genes of broiler chickens in response to variable dietary total Ca and phytase supplementation are influenced by time on experimental diets and age of the birds. Poult. Sci..

[bib0030] Yu S., Cowieson A., Gilbert C., Plumstead P., Dalsgaard S. (2012). Interactions of phytate and myo-inositol phosphate esters (IP_1-5_) including IP_5_ isomers with dietary protein and iron and inhibition of pepsin. J. Anim. Sci..

[bib0031] Zhang Q., Walk C., Sorbara J.-O.B., Cowieson A.J., Stamatopoulos K. (2022). Comparative effects of two phytases on growth performance, bone mineralization, nutrient digestibility, and phytate-P hydrolysis of broilers. J. Appl. Poult. Res..

